# Unique patterns of glycosylation in immunoglobulin subclass G4‐related disease and primary sclerosing cholangitis

**DOI:** 10.1111/jgh.14512

**Published:** 2018-11-22

**Authors:** Emma L Culver, Fleur S van de Bovenkamp, Ninotska I L Derksen, Jana Koers, Tamsin Cargill, Eleanor Barnes, Louise A de Neef, Carolien A M Koeleman, Rob C Aalberse, Manfred Wuhrer, Theo Rispens

**Affiliations:** ^1^ Sanquin Research, Department of Immunopathology, and Landsteiner Laboratory, Academic Medical Centre University of Amsterdam Amsterdam The Netherlands; ^2^ Translational Gastroenterology Unit and Oxford NIHR Biomedical Research Centre John Radcliffe Hospital Oxford UK; ^3^ Center for Proteomics and Metabolomics Leiden University Medical Center Leiden The Netherlands

**Keywords:** complement, glycosylation, IgG4 antibody, IgG4‐related disease, primary sclerosing cholangitis

## Abstract

**Background and Aim:**

Immunoglobulin subclass G4‐related disease (IgG4‐RD) is characterized by an abundance of IgG4 antibodies in the serum and tissue. Glycosylation status of antibodies can impact on immune effector functions and disease pathophysiology. We sought to establish glycosylation patterns in a prospective cohort of patients with IgG4‐RD and the relationship with disease activity and response to treatment.

**Methods:**

We assessed IgG Fc‐tail and Fab‐arm glycosylation status in patients with IgG4‐RD (*n* = 22), disease controls with primary sclerosing cholangitis (PSC) (*n* = 22), and healthy controls (*n* = 22). Serum IgG and subclasses were quantified using ELISA. Fc and Fab glycosylation were analyzed by mass spectrometry and lectin affinity chromatography, respectively. Disease activity, organ damage, and response to treatment were assessed using the IgG4 Responder Index.

**Results:**

Immunoglobulin G Fab sialylation was increased in IgG4‐RD compared with PSC and healthy control (*P* = 0.01), with a preferential increase in IgG4‐specific Fab sialylation, which was independent of IgG4 Fab‐arm exchange. There was a reduction in IgG1‐specific Fc bisection and hybrid structures in IgG4‐RD (*P* < 0.01), which recovered upon steroid treatment and correlated with disease activity. Overall, IgG Fc galactosylation was reduced in both IgG4‐RD and PSC (*P* < 0.01), with a preferential reduction in IgG1‐specific sialylation and enhancement of IgG4‐specific bisection in PSC. IgG4 fucosylation and IgG1/2/3 hybrid structures negatively correlated with complement C3 and C4 levels in IgG4‐RD (*P* < 0.01), but not PSC.

**Conclusion:**

We report the first study showing unique antibody glycosylation status in a prospective cohort of IgG4‐RD and PSC patients, which may determine modulation of the immune system and contribute to disease pathophysiology.

## Introduction

Immunoglobulin subclass G4‐related disease (IgG4‐RD) is a systemic fibro‐inflammatory condition, characterized by abundant IgG4^+^ polyclonal plasma cells, with organ dysfunction related to the tumor‐like mass of these cells.^1^ IgG4‐related sclerosing cholangitis and autoimmune pancreatitis are the biliary and pancreatic manifestations of this disease. A role for IgG4 (auto) antibodies in disease pathogenesis is supported by three observations: (i) oligoclonal expansion of IgG4^+^‐dominant B‐cell receptor (BCR) clones in the blood and tissue of patients with active IgG4‐RD^2^; (ii) the identification of IgG4 autoantibodies (annexin A11‐specific) in a proportion of IgG4‐RD patients^3^; and (iii) B‐cell depletion therapy with rituximab, which attenuates disease activity and selectively depletes the IgG4 subclass.^4^ Furthermore, a recent study demonstrated pancreatic injury in BALB/c mice induced by injection of IgG4 antibodies purified from patients with autoimmune pancreatitis, although more destructive changes were exhibited by injection of IgG1 antibodies, and IgG4 inhibited the pathogenic effects of IgG1 on simultaneous injection.^5^

The glycosylation pattern of an antibody crucially influences its conformation, aggregation behavior, and ability to bind to Fc gamma receptors (FcγR) and complement, thus its cellular effector functions.^6^, ^7^ IgG4 antibodies are themselves unique: They exhibit weak binding to low‐affinity FcγRs, do not activate the classical complement pathway, and undergo Fab‐arm half‐molecule exchange resulting in asymmetric bi‐specific antibodies that are unable to form large immune complexes.^8^, ^9^

We recently demonstrated that glycosylation of the Fab arms of IgG4 antibodies is increased compared with that of the other IgG subclasses.^10^ Furthermore, increased IgG (auto) antibody activity has been associated with a higher pathogenicity of autoantibodies in systemic lupus erythematosus, rheumatoid arthritis (RA), and inflammatory bowel disease and with disease severity in systemic lupus erythematosus and RA.^11^, ^12^ Lower glycosylation results in a more pronounced immune response, although complete removal of the Fc sugar residues abrogates IgG activity. In contrast, enrichment of sialylation by therapeutic intravenous immunoglobulin (IVIg) can be utilized to ameliorate pro‐inflammatory effects.^13^

Whether a characteristic glycosylation pattern confers novel effector function of lgG4 antibodies and/or preferential recruitment and retention of IgG4‐switched B cells into the affected tissue in patients with IgG4‐RD is unknown. This is the first study to investigate Fc and Fab antibody glycosylation patterns in patients with IgG4‐RD and investigate the relationship of disease activity, organ damage, and treatment with glycosylation status.

## Methods

### Recruitment of patients and controls

Immunoglobulin subclass G4‐RD patients (*n* = 22 with biliary disease, and other organ involvement), disease controls (DCs) (*n* = 22 with primary sclerosing cholangitis [PSC]), and healthy controls (HCs) (*n* = 22) were prospectively recruited from the John Radcliffe Hospital, Oxford, UK, a tertiary referral center for IgG4‐RD and PSC. Patients enrolled in this study had serum collected at diagnosis before treatment (IgG4‐RD and PSC) and after 6–8 weeks of corticosteroid treatment (IgG4‐RD) and were prospectively followed‐up. Ethical approval for the study was obtained from the Research Ethics Committee Oxfordshire (10/H0604/51), and the study was registered on the UK National Institute of Health Research portfolio (10776).

### Diagnostic criteria

The diagnosis of IgG4‐RD was made using the HISORt criteria (2006/7) for IgG4‐related pancreatic and biliary disease^14^ and the Japanese Comprehensive Diagnostic Criteria (2011) for systemic disease.^15^ Organ involvement included the bile ducts (22), pancreas (18), salivary glands (6), lacrimal glands (2), lymph nodes (3), lung (5), kidney (2), mesentery (3), retroperitoneum (1), and aorta (1).

Disease controls were patients with PSC, diagnosed in accordance with the EASL guidelines for cholestatic liver disease.^16^ PSC patients were chosen as DC's as both IgG4‐RD and PSC share an immune‐mediated etiology, present with a similar gender and age distribution, have elevated serum IgG4 levels in a proportion of patients, and have biliary tree involvement. HCs had no known immune or inflammatory disease.

### Clinical data collection

Clinical data were entered onto IgG4‐RD Registry database https://igg4rd.oxnet.nhs.uk for IgG4‐RD and the Oxford ACCESS database for PSC patients. Data recorded in IgG4‐RD patients included baseline demographics, serological evaluation at diagnosis, remission and relapse, imaging characteristics, histological morphology and immunostaining, organ involvement, disease activity and organ damage, treatment and response, disease relapse, malignancy, and mortality.

### Immunoglobulin subclass G4 Responder Index

Immunoglobulin subclass G4‐RD activity, organ damage, and response to treatment were assessed using the IgG4 Responder Index (IgG4‐RI).^17^ This assessment tool has recently been validated in an international multi‐speciality study of IgG4‐RD for assessment of disease activity and longitudinally for treatment response.^18^ Scoring for disease activity (present in the preceding 28 days) and organ damage is given for 24 standard organ/sites, incorporating higher weights for disease manifestations that urgently require treatment or that worsen despite treatment. Disease activity reflects a patient's symptoms attributable to active IgG4‐RD as well as significant findings from the physical examination, imaging studies, and laboratory evaluations. Organ damage refers to irreversible organ dysfunction (e.g. exocrine pancreatic insufficiency) or failure (e.g. chronic kidney disease) caused by IgG4‐RD or as a consequence of surgical interventions performed to diagnose or treat it (e.g. Whipple procedures, submandibular gland excisions).

### Immunoglobulin G and immunoglobulin subclass G4 nephelometry

Serum IgG and subclasses were measured at diagnosis and during treatment (remission and relapse). Comparisons between IgG4‐RD patients, DCs, and HCs were made at diagnosis, before steroid initiation. Total serum IgG and IgG4 were measured by nephelometry (BNII analyzer, Siemens, UK). Elevated serum IgG (≥ 16 g/L) and IgG4 (≥ 1.4 g/L) were defined by institution range (Immunology Department, Churchill Hospital, Oxford).

### Complement levels

Complement levels were measured at diagnosis in IgG4‐RD patients and on recruitment in PSC patients. C3 and C4 levels were measured by turbidimetry. Reduced C3 (< 65 mg/dL and C4 < 14 mg/dL) levels were defined by institution range (Immunology Department, Churchill Hospital, Oxford).

### Immunoglobulin G and immunoglobulin G subclass ELISA

Human IgG was quantified using an ELISA as described previously.^10^ In short, microtiter plates were coated with monoclonal mouse antihuman IgG, incubated with samples, and detected with horseradish peroxidase (HRP)‐conjugated monoclonal mouse antihuman IgG and tetramethylbenzidine substrate solution.

Human IgG subclass ELISAs were carried out analogously to the IgG ELISA. Microtiter plates were coated with monoclonal mouse antihuman IgG1 or IgG4, incubated with samples, and detected with horseradish peroxidase hepatobiliary‐conjugated polyclonal rabbit antihuman IgG and tetramethylbenzidine substrate solution.

### Mass spectrometry analysis

Fc glycosylation was analyzed by mass spectrometry as described previously.^19^ In short, IgG was affinity‐purified using protein G beads, trypsinized, and resulting IgG Fc‐glycopeptides were analyzed by nano‐LC–MS using an ACQUITY BEH C18 column (130 Å, 1.7 μm; 75 μm × 100 mm; Waters, Milford, MA) for glycopeptide separation. Mass spectrometric signals of glycopeptides were processed and integrated using LacyTools^19^ and normalized per IgG subclass to 100%, and levels of sialylation, galactosylation, bisection, fucosylation, and hybrid structures were calculated.

### Recombinant immunoglobulin subclass G4 antibodies

Recombinant IgG4 antibodies (adalimumab [ADL] wild‐type without Fab glycans and ADL mutant D86N with Fab glycans) were produced using the serum‐free FreeStyle 293 expression system (Invitrogen) as described previously.^10^ During transfection, sialyltransferase (ST6GALT) and D‐galactose were added to the medium.^20^ After culture, antibodies were purified using protein G Sepharose. *Sambucus nigra* agglutinin (SNA) western blot confirmed sialylation of Fab and Fc glycans in recombinant IgG4 antibodies to a degree similar or higher compared with polyclonal IVIg (data not shown). The *N*‐glycosylation site in the Fab arm of ADL mutant D86N was selected and introduced as described previously.^10^

### Immunoglobulin subclass G4 half‐molecule exchange

Immunoglobulin subclass G4 ADL wild‐type (without Fab glycans) and IgG4 ADL mutant D86N (with Fab glycans) were incubated in a 4:1 ratio in 1‐mM glutathione for 24 h at 37°C while shaking to allow IgG4 half‐molecule exchange to take place.^21^ To stop the reaction, 2‐mM 2‐iodoacetamide was used. Exchanged antibodies were dialyzed overnight against Tris‐buffered saline.

### Lectin (*Sambucus nigra* agglutinin) affinity chromatography

Fab glycosylation was analyzed by lectin (SNA) affinity chromatography as described previously.^10^ Using this technique, essentially only sialylated Fab glycans (~90% of total Fab glycans^22^), but not sialylated Fc glycans,^23^ are retained. In short, serum samples were applied to a Tricorn column containing SNA agarose. After washing away unbound proteins (SNA‐depleted fraction), bound proteins (SNA‐enriched fraction) were eluted with 0.5 M lactose in 0.2 M acetic acid and immediately dialyzed against phosphate‐buffered saline. In case of recombinant IgG4 antibody samples, buffers were supplemented with 0.05% polysorbate‐20. The percentage of Fab glycosylated antibodies was calculated by dividing the amount of IgG in the SNA‐enriched fraction by the amount of IgG in the SNA‐depleted and SNA‐enriched fractions as measured by the ELISAs described previously.

## Results

### Serum total immunoglobulin G and immunoglobulin subclass G4 levels

**Figure 1:**
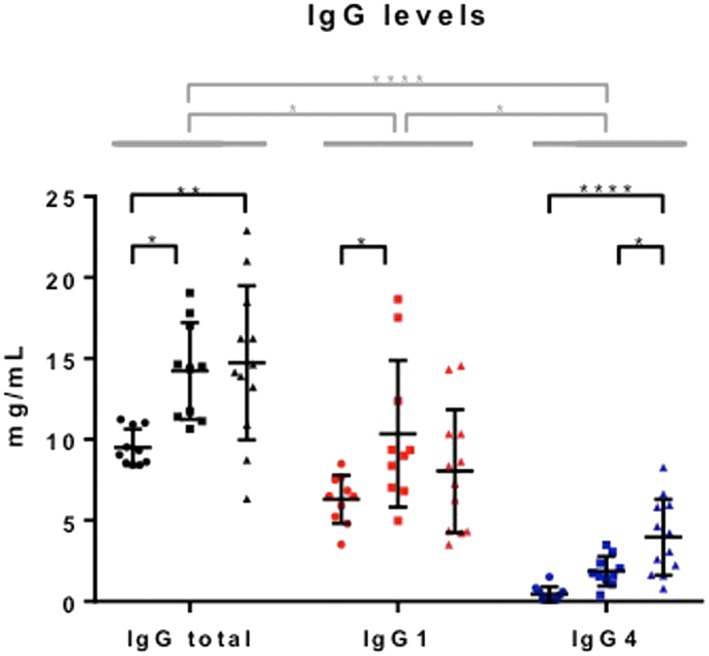
Serum immunoglobulin G (IgG) and subclass levels in immunoglobulin subclass G4‐related disease (IgG4‐RD) patients and controls. Levels of IgG total, IgG1, and IgG4 in healthy controls (HCs, *n* = 10), disease controls (DCs, *n* = 10), and IgG4‐RD patients (*n* = 9–12) measured using ELISA. Means with SDs are represented. Ordinary one‐way ANOVA with Tukey's multiple comparison test, *P < 0.05, **P < 0.01, ***P < 0.001, ****P < 0.0001. , HCs; , DCs; , IgG4‐RD. [Color figure can be viewed at http://wileyonlinelibrary.com]

Patients with IgG4‐RD (male 73% [16/22]; median age 64.3 [range 33–84] years), PSC (male 77% [17/22]; median age 59.9 [range 33–77] years), and HCs (male 73% [16/22]; median age 58.1 [range 33–78] years) were well matched (*P* = NS). Serum total IgG levels were higher in IgG4‐RD and PSC patients compared with HCs (14.7 *vs* 14.2 *vs* 9.5 mg/mL; IgG4‐RD *vs* HCs *P* = 0.004 and DCs *vs* HCs *P* = 0.012) (Fig. 1, left). Serum IgG1 levels were higher in PSC patients compared with HCs (10.4 *vs* 6.3 mg/mL; *P* = 0.04) only (Fig. 1, middle). Serum IgG4 levels in IgG4‐RD patients were higher compared with PSC and HCs (4.0 *vs* 1.9 *vs* 0.4 mg/mL; IgG4‐RD *vs* DCs *P* = 0.01 and IgG4‐RD *vs* HCs *P* < 0.0001) (Fig. 1, right).

Serum IgG4 levels fell with corticosteroid treatment in IgG4‐RD patients (*P* < 0.0001) (Fig. S1a) and correlated with disease activity (*P* = 0.005) (Fig. S1b) but not organ damage (*P* = 0.21), using the IgG4‐RI. This was not the case for the other subclasses (data not shown).

### Immunoglobulin G Fab glycosylation

**Figure 2:**
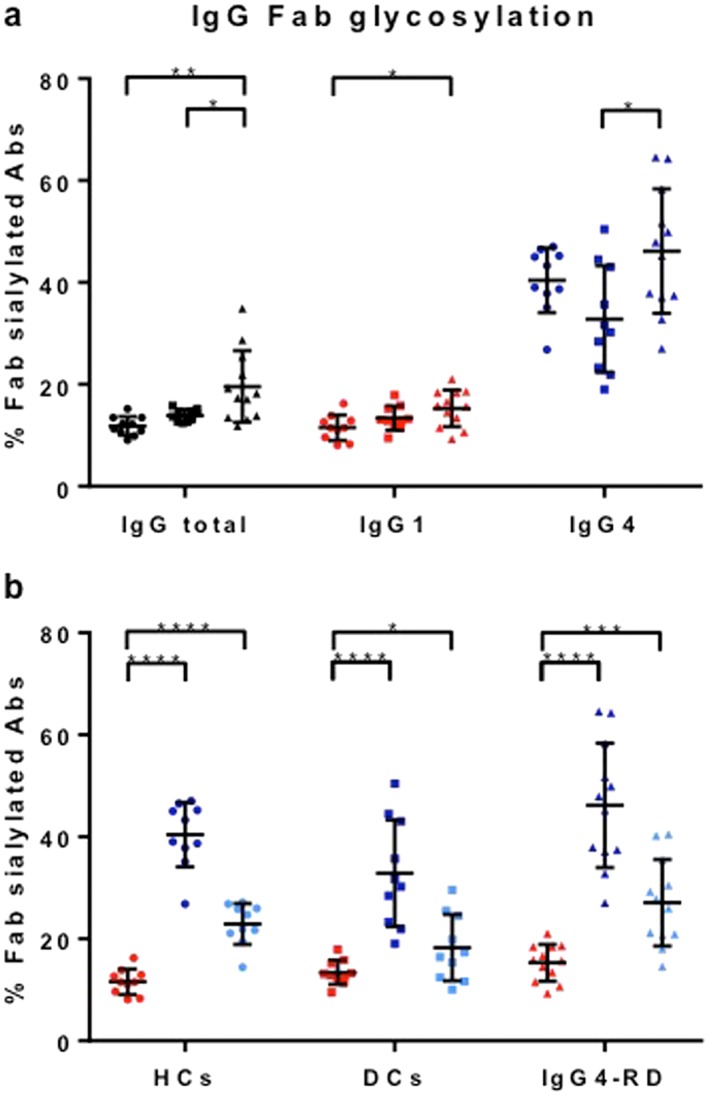
Immunoglobulin G (IgG) Fab glycosylation in immunoglobulin subclass G4‐related disease (IgG4‐RD) patients and controls. Percentage of Fab glycosylation in healthy controls (HCs, *n* = 8–10), disease controls (DCs, *n* = 7–10), and IgG4‐RD patients (*n* = 7–12) for IgG total, IgG1, and IgG4 estimated by determining IgG fractions with/without Fab sialylation using *Sambucus nigra* agglutinin affinity chromatography and ELISA shown per group (a; , HCs; , DCs; , IgG4‐RD) or subclass (b; IgG1; left, IgG4; middle, IgG4 corrected right). For IgG4, percentages are also shown recalculated to exclude the effect of half‐molecule exchange (Fig. 3). Means with SDs are represented. Ordinary one‐way ANOVA with Tukey's multiple comparison test, *P < 0.05, **P < 0.01, ***P < 0.001, ****P < 0.0001. [Color figure can be viewed at http://wileyonlinelibrary.com]

Fab glycosylation levels were estimated for IgG4‐RD patients (*n* = 12 with active disease), PSC patients (*n* = 10), and HCs (*n* = 10) using SNA affinity chromatography, whereby essentially only sialylated Fab glycans are retained. Total IgG Fab sialylation was enhanced in IgG4‐RD compared with PSC and HCs (*P* < 0.05; Fig. 2a, left). IgG4‐specific Fab sialylation was preferentially higher in IgG4‐RD compared with PSC (*P* = 0.01; Fig. 2a, right), and IgG1‐specific Fab sialylation was higher in IgG4‐RD compared with HCs (*P* = 0.01; Fig. 2a, middle). These findings were partly attributed to the higher levels of total IgG and IgG4 seen in IgG4‐RD patients compared with PSC and HC groups (Fig. 1).

**Figure 3:**
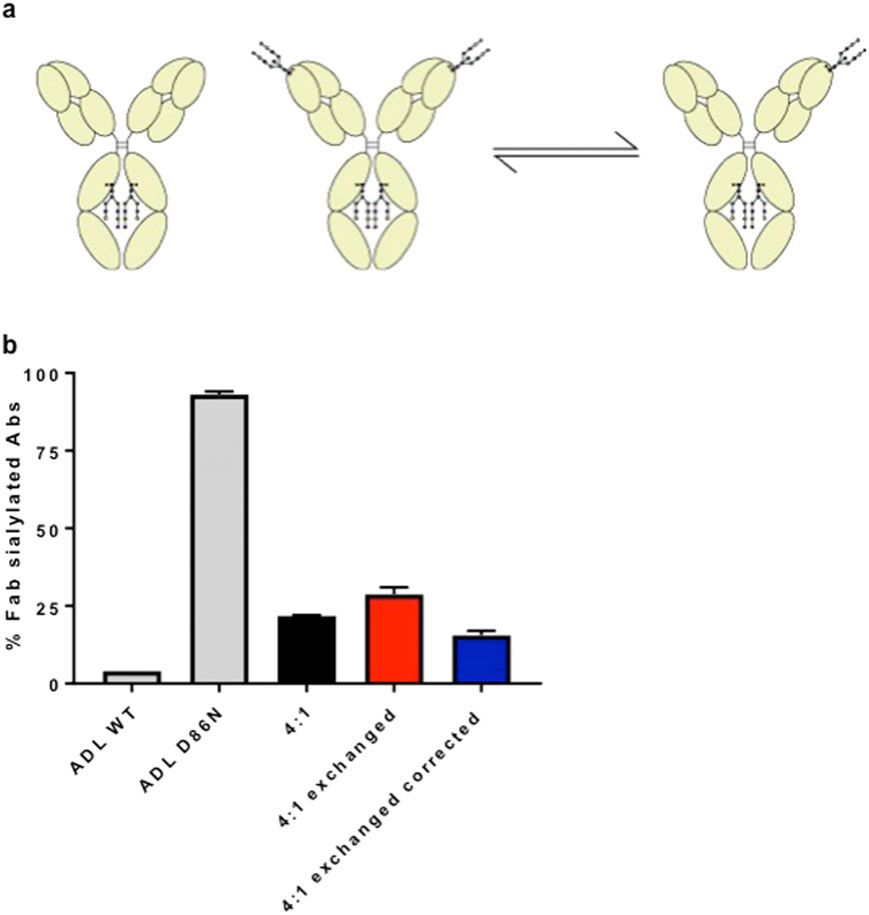
The effects of half‐molecule exchange on the retention of immunoglobulin G (IgG) with and without Fab glycosylation during *Sambucus nigra* agglutinin (SNA) affinity chromatography. (a) Schematic representation of immunoglobulin subclass G4 (IgG4) half‐molecule exchange. Under the premise that all antibodies carrying at least one sialylated Fab glycan will be retained on an SNA column, the total number of IgG4 molecules bound will be larger upon half‐molecule exchange, in case of a mixture of antibodies with/without Fab glycans. (b) Percentages of Fab sialylated antibodies (Abs) for IgG4 adalimumab (ADL) wild‐type (WT, without Fab glycans), IgG4 ADL mutant (D86N, with Fab glycans), and a 4:1 mixture of those before (black bar) and after (red bar) incubation in glutathione and corrected for IgG4 half‐molecule exchange (blue bar), as measured by ELISA after SNA affinity chromatography and calculated as described in Methods. Means of two replicates are shown with SEMs. [Color figure can be viewed at http://wileyonlinelibrary.com]

Overall, we observed an increase in IgG4‐specific Fab glycosylation in all three groups (IgG4‐RD patients, PSC, and HCs) compared with that of the other IgG subclasses (*P* < 0.0001; Fig. 2b). This result can partially be explained by the ability of IgG4 antibodies to engage in half‐molecule exchange, resulting in an increased fraction of IgG4 antibodies that carry at least one glycan^10^ (Fig. 3a). Therefore, percentages are also shown recalculated to non‐exchanged IgG4 and remain elevated (Fig. 2b). Additionally, we also tested if exchange of monoclonal IgG4 with/without Fab glycans would result in retention exceeding that expected on the basis of molar ratio alone. Indeed, a 4:1 mixture of recombinant IgG4 antibodies without/with Fab glycans resulted in about 29% retention *versus* 20% if half‐molecule exchange does not occur and 36% if half‐molecule exchange does occur (Fig. 3b).

### Immunoglobulin G Fc glycosylation patterns

**Figure 4:**
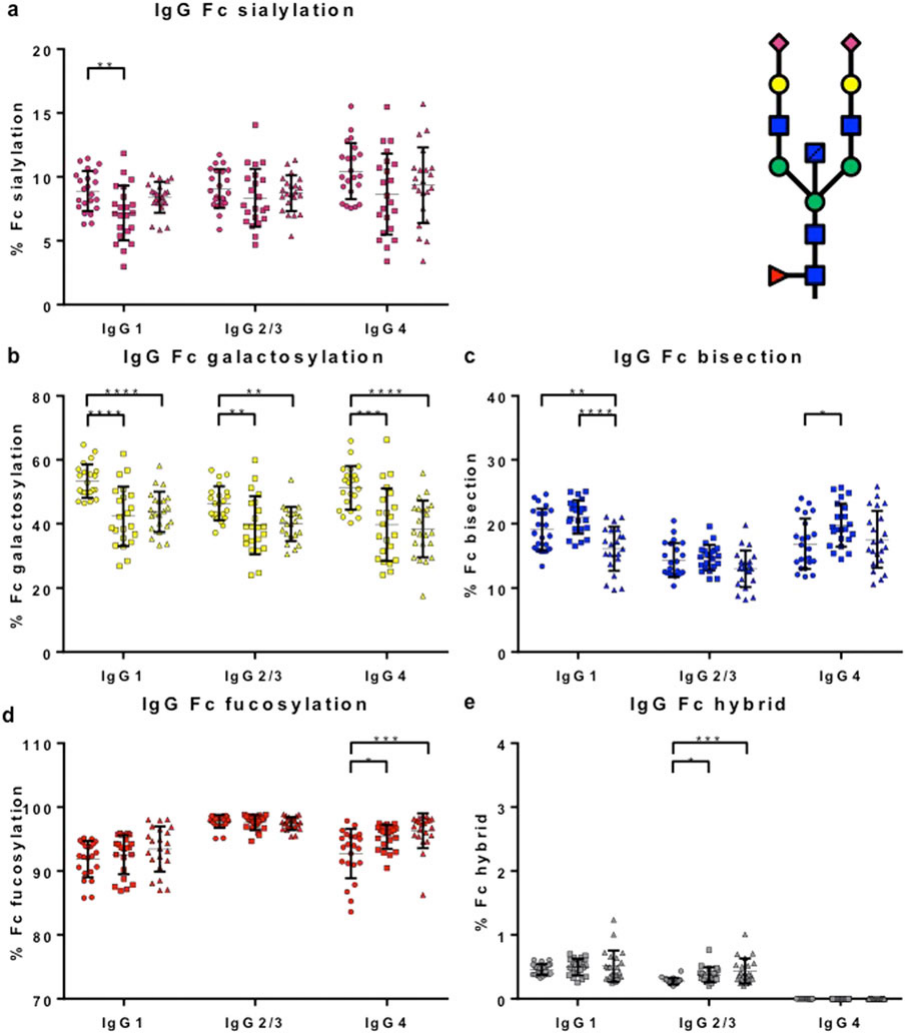
Immunoglobulin G (IgG) Fc galactosylation is decreased in immunoglobulin subclass G4‐related disease (IgG4‐RD) and primary sclerosing cholangitis patients. Percentage of IgG Fc (a) sialylation, (b) galactosylation, (c) bisection, (d) fucosylation, and (e) hybrid structures (one branch with mannose residues and one branch with complex residues) in healthy controls (HCs, *n* = 22), disease controls (DCs, *n* = 22), and IgG4‐RD patients (*n* = 22) per subclass (IgG1, IgG2/3 [not individually resolved], and IgG4) measured using mass spectrometry. Means with SDs are represented. Ordinary one‐way ANOVA with Tukey's multiple comparison test, *P < 0.05, **P < 0.01, ***P < 0.001, ****P < 0.0001. , HCs; , DCs; , IgG4‐RD. [Color figure can be viewed at http://wileyonlinelibrary.com]

Immunoglobulin subclass G1, IgG2/3 and IgG4 Fc glycosylation patterns (sialylation, galactosylation, bisection, fucosylation, and hybrid structures) were determined for patients (IgG4‐RD *n* = 22 with active disease) and controls (PSC *n* = 22; HCs *n* = 22) using mass spectrometry. Fc glycosylation patterns in HCs were in accordance with recently published data (Fig. 4a–e).^24^

Immunoglobulin G Fc galactosylation was decreased in IgG4‐RD and PSC patients compared with HCs (*P* < 0.01) (Fig. 4b), a finding that has been seen in other autoimmune and chronic inflammatory conditions. There was an increase in IgG4‐specific Fc fucosylation (Fig. 4d) and hybrid structures in IgG2/3 Fc (Fig. 4e) in IgG4‐RD and PSC patients compared with HCs (*P* < 0.05).

**Figure 5:**
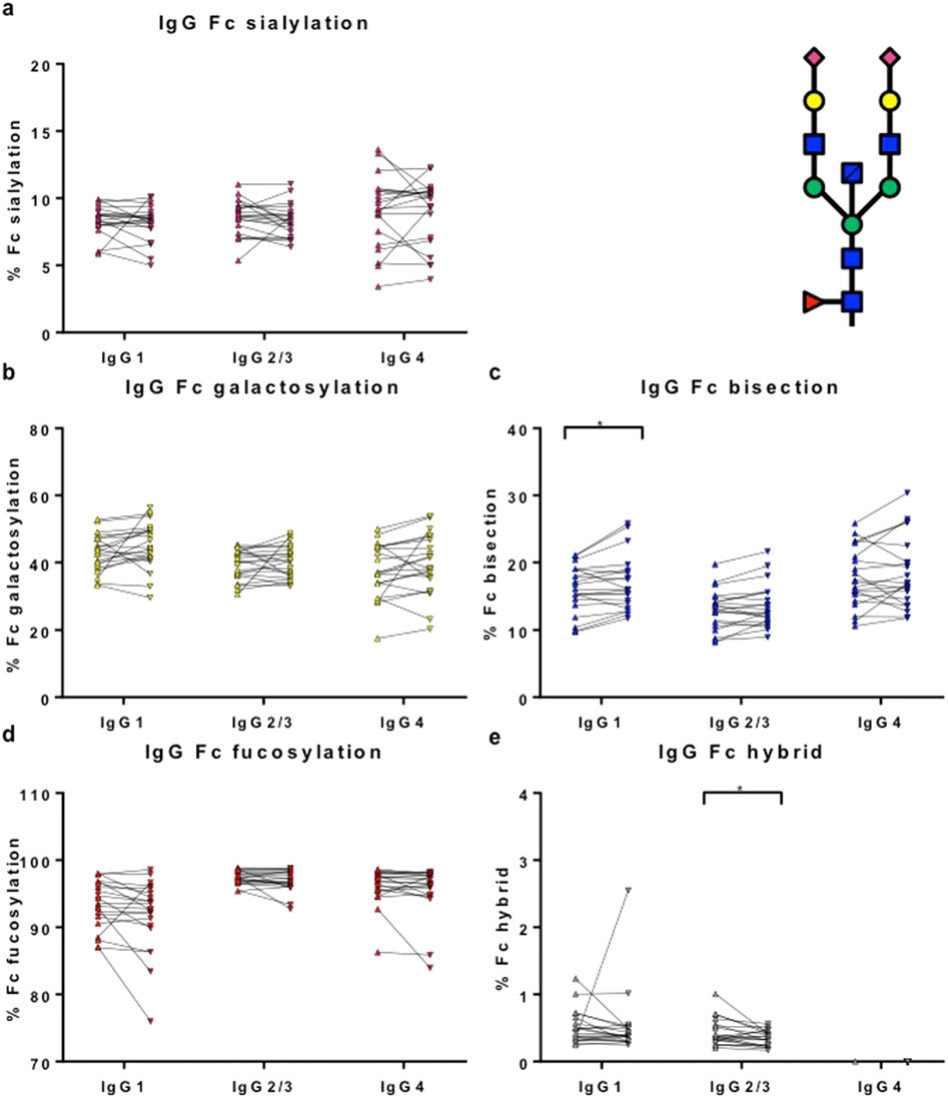
Immunoglobulin G (IgG) Fc glycosylation in immunoglobulin subclass G4‐related disease (IgG4‐RD) patients before and after treatment. Percentage of IgG Fc (a) sialylation, (b) galactosylation, (c) bisection, (d) fucosylation, and (e) hybrid structures (one branch with mannose residues and one branch with complex residues) in IgG4‐RD patients before (pre, *n* = 21) and after (post, *n* = 21) treatment per subclass (IgG1, IgG2/3, and IgG4) measured using mass spectrometry. Paired *t*‐test, *P < 0.05. 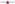, IgG4‐RD pre; 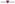, IgG4‐RD post. [Color figure can be viewed at http://wileyonlinelibrary.com]

Interestingly, IgG1‐specific Fc bisection was decreased in IgG4‐RD patients exclusively, when compared with PSC patients and HCs (*P* < 0.01) (Fig. 4c) and recovered with corticosteroid treatment (Fig. 5c). In contrast, IgG4‐specific Fc bisection was increased (Fig. 4c) and IgG1‐specific Fc sialylation was decreased (Fig. 4a) in PSC patients compared with HC, with no difference seen in IgG4‐RD.

### Recovery of glycosylation status with treatment

Fc glycosylation patterns were determined for IgG4‐RD patients before (pre) and after (post 6–8 weeks) corticosteroid treatment (*n* = 21), to investigate whether the observed differences in Fc glycosylation are recovered (Fig. 5a–e). There was recovery of IgG1‐specific Fc bisection (increase; *P* = 0.04; Fig. 5c) and IgG2/3 Fc hybrid structures (decrease; *P* = 0.02; Fig. 5e) upon treatment of IgG4‐RD patients. In line with this, IgG1‐specific Fc bisection and IgG2/3 Fc hybrid structures were decreased and increased compared with HCs, respectively (Fig. 4).

### Correlation of glycosylation status with disease activity and organ damage

Fc glycosylation patterns were assessed with respect to disease activity and organ damage, using the IgG4‐RI in IgG4‐RD. The IgG4‐RI total activity score fell in response to corticosteroid therapy (*P* < 0.0001) (Fig. S2a). IgG2/3 Fc hybrid structures correlated with the IgG4‐RI (*P* < 0.01) (Fig. S2b), indicating that an increase in IgG2/3 Fc hybrid structures is associated with more active disease and may serve as a disease biomarker. There was no alteration in glycosylation status in patients with organ damage (fibrotic) (data not shown).

### Correlation of glycosylation status with complement levels

Fc glycosylation patterns were assessed with respect to complement levels in IgG4‐RD and PSC. C3 and/or C4 levels were reduced in 5 of 22 (23%) IgG4‐RD (median C3 126 mg/dL; C4 25.9 mg/dL) and in 1 of 12 (8%) PSC patients (median C3 115 mg/dL; C4 20.9 mg/dL) when measured at baseline. The patient with hypocomplementaemia in the PSC group had co‐existent Sjogren's syndrome. IgG4 fucosylation and IgG1/2/3 hybrid structures negatively correlated with C3 and C4 levels in IgG4‐RD (Fig. S3a–f). In PSC patients, there was no correlation of C3/C4 with glycosylation status (data not shown).

## Discussion

In the first study to investigate IgG glycosylation in patients with IgG4‐RD and PSC, we report unique glycosylation patterns in patients with active disease and in response to treatment. Specifically, we observed enhancement of IgG and IgG4‐specific Fab sialylation in IgG4‐RD compared with PSC and HC. Furthermore, there was a decrease in IgG Fc galactosylation and IgG1‐specific Fc bisection in those with active IgG4‐RD compared with HC, with a recovery of IgG1‐specific bisection with treatment. There was also an increase in IgG4‐specific Fc fucosylation and Fc hybrid structures, the latter responded to treatment and correlated with disease activity.

Fab glycans are complex‐type biantennary *N*‐glycans linked to *N*‐glycosylation sites that emerge during somatic hypermutation. IgG Fab glycosylation is an important process in immunity, with demonstrated effects on stability, half‐life, and binding characteristics of antibodies and BCR.^10^, ^25^ Given the abundance of IgG4 antibodies in the serum and involved organs of patients with IgG4‐RD and a subset of those with PSC, we considered it plausible that Fab glycosylation might contribute to this. Indeed, we showed that IgG Fab sialylation was enhanced in IgG4‐RD patients and that IgG4‐specific Fab sialylation was preferentially enhanced in IgG4‐RD compared with PSC patients and HC, irrespective of Fab‐arm exchange and higher than expected on the basis of molecular ratio alone. This supports the concept that IgG4 antibodies have a unique effector function in IgG4‐RD, perhaps interacting with Siglecs, which has been suggested for therapeutic IVIg.^26^ Furthermore, endogenous or exogenous lectins may interact with glycans to exert immunomodulatory effects, as seen in follicular lymphoma B cells (stimulated through the interaction between a mannose‐binding lectin and high‐mannose structures at the IgG4 Fab of their BCRs), which may also be the case for IgG4 antibodies in IgG4‐RD.^27^

Immunoglobulin glycosylation plays a crucial role in modulating antibody‐mediated responses and skewing the immune system toward a pro‐inflammatory or anti‐inflammatory direction. Reduction in total serum and antigen‐specific IgG Fc galactosylation is one of the most prominent changes observed in a wide variety of chronic inflammatory and autoimmune diseases, believed to be important in complement activation and antibody‐mediated phagocytosis.^28^ We similarly observed this phenomenon in IgG4‐RD and PSC patients with active disease. Terminal sialylation of the Fc of IgG controls its anti‐inflammatory and/or pro‐inflammatory effects. We observed decreased IgG1‐specific Fc sialylation in PSC patients, supporting increased cytotoxicity and pro‐inflammatory effects of IgG in active disease. The state of IgG‐fucosylation is implicated in antibody‐dependent cellular cytotoxicity and FcR effector functions.^29^ We observed increased IgG4‐specific Fc fucosylation in both IgG4‐RD and PSC. Non‐fucosylated IgG1 (and IgG4) antibodies bind FcγRIIIa with a 20–30‐fold higher affinity than fucosylated antibodies, and the small increase in fucosylation is in fact an almost twofold decrease in the relative amount of these non‐fucosylated antibodies.^30^ This interesting observation may suggest novel FcR binding and reduced antibody‐dependent cytotoxicity in patients with IgG4‐RD and PSC.

Agalactosylated N‐linked glycans (G0 N‐glycan) have been suggested to represent a more pro‐inflammatory phenotype, for example, with respect to activation of complement pathways. Hypocomplementaemia has been described in patients with active IgG4‐RD.^31^, ^32^ In this study, C3 and/or C4 levels were reduced in 23% of 22 IgG4‐RD patients. In a US cohort, reduced C3 (20%), C4 (19%), or both (11%) were present in 103 IgG4‐RD patients and were associated with a more inflammatory phenotype, higher serum IgG4 levels, and IgG4‐related kidney involvement.^32^ Recently, a small study reported increased IgG4 G0 N‐glycans and IgG4 fucosylated N‐glycans in patients with IgG4‐RD (*n* = 12) compared with HCs (*n* = 8), with IgG4 non‐fucosylated N‐glycans decreased in those with hypocomplementaemia (*n* = 7).^33^ In line with this, we found a negative correlation between C3 and C4 levels and both IgG4 fucosylation, and IgG1/2/3 hybrid structures. The relevance of this remains uncertain, given that fucosylation and galactosylation are primary mediators of functional changes in IgG for FcγR‐mediated and complement‐mediated effector functions, respectively.^7^

Recent studies have demonstrated that abnormalities in IgG glycosylation play a major role not only in triggering but also in the activity and relapses in autoimmune disease.^34^ For example, in patients with vasculitis, low‐sialylated PR3‐ANCA autoantibodies are observed in those with high disease activity and serve as a biomarker of disease.^34^ Furthermore, recovery of glycosylation status can be seen in response to treatment, for example, in RA patients treated with methotrexate.^35^ We observed a decrease in IgG1‐specific Fc bisection and recovery (increased) with corticosteroid treatment specifically in IgG4‐RD patients compared with PSC and HC. This may be interesting to explore as a biomarker of active disease. However, mass spectrometry is not widely available, and thus, technological advancements in the use of plant lectins (with highly specific carbohydrate domains) would be required to detect functionally relevant alterations of glycosylation patterns in this disease.

Taking into account the central role of glycosylation, it could be considered an attractive target for the development of safer therapy in patients with IgG4‐RD and PSC. B‐cell depletion is an attractive therapeutic option in IgG4‐RD. Non‐fucosylated recombinant anti‐CD20 rituximab (BLX‐300) appears to be as efficient as classical fucosylated rituximab in target‐cell binding and apoptosis induction but is considered to be less toxic in *in vitro* studies.^36^ Such an approach could improve the safety of therapeutic monoclonal antibodies without diminishing their efficiency. Interestingly, treatments based on glycosylation modulation could might be also efficient in allergic diseases, which are over‐represented in patients with IgG4‐RD.^37^ For instance, in an OVA‐induced allergic airway inflammation mouse model, prophylactic treatment based on sialylated anti‐OVA IgG transfer could inhibit IgG and IgE anti‐OVA synthesis, plasma cell development, and lung eosinophil infiltration.^38^ In summary, a better understanding of the role and the mechanisms of immunoglobulin glycosylation alterations in IgG4‐RD may pave the way for the future development of safer and more innovative treatments.

## Supporting information


**Figure S1.** Serum IgG4 levels with disease activity and corticosteroid treatment in IgG4‐RD. (a) Correlation plot of serum IgG4 levels with total disease activity calculated using the IgG4‐Responder Index. Spearman rank correlation, ^**^ *P* < 0.01. (b) Paired data of serum IgG4 levels pre and post corticosteroid treatment. Wilcoxen signed rank test, ^****^ *P* < 0.0001.

**Figure S2.** IgG4‐Responder Index (IgG4‐RI) activity score with corticosteroid therapy and glycosylation status. (a) Paired data of IgG4‐RI activity score pre and post corticosteroid treatment. Paired t test, ^****^ *P* < 0.0001. (b) Correlation plot of the IgG4‐RI activity score and IgG2/3 Fc hybrid structure glycosylation. Linear Regression Analysis, ^**^ *P* < 0.01.

**Figure S3.** Complement levels with glycosylation status in IgG4‐RD. Correlation plot of C3 levels with (a) fucosylation, and (b) IgG1 hybrid structures (c) IgG2/3 hybrid structures. Correlation plot of C4 levels with (d) fucosylation, and (e) IgG1 hybrid structures (f) IgG2/3 hybrid structures. Spearman rank correlation, ^*^ *P* < 0.05 ^**^ *P* < 0.01.
