# Supplementary figures and images for: Unique patterns of glycosylation in immunoglobulin subclass G4‐related disease and primary sclerosing cholangitis
Source: J Gastroenterol Hepatol. 2018 Nov 22;34(10):1878–86. doi: 10.1111/jgh.14512 (PMC6899843; doi:10.1111/jgh.14512)

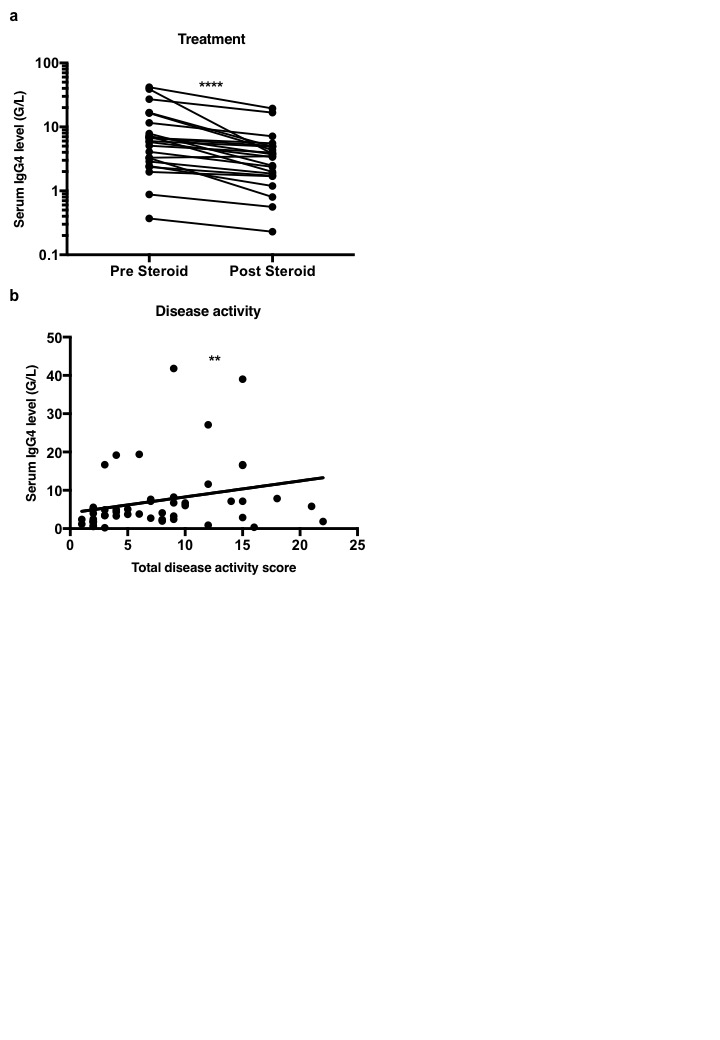

Supplement: Supplementary file 1 — Figure S1. Serum IgG4 levels with disease activity and corticosteroid treatment in IgG4‐RD. (a) Correlation plot of serum IgG4 levels with total disease activity calculated using the IgG4‐Responder Index. Spearman rank correlation, ** P < 0.01. (b) Paired data of serum IgG4 levels pre and post corticosteroid treatment. Wilcoxen signed rank test, **** P < 0.0001. [file JGH-34-1878-s001.jpg]

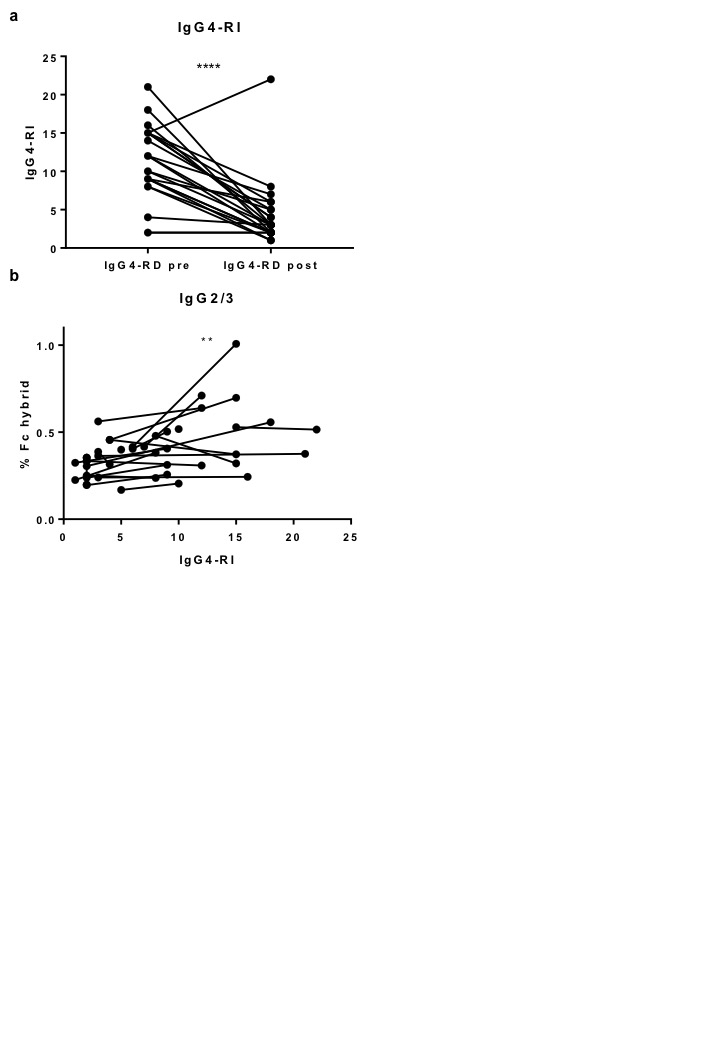

Supplement: Supplementary file 2 — Figure S2. IgG4‐Responder Index (IgG4‐RI) activity score with corticosteroid therapy and glycosylation status. (a) Paired data of IgG4‐RI activity score pre and post corticosteroid treatment. Paired t test, **** P < 0.0001. (b) Correlation plot of the IgG4‐RI activity score and IgG2/3 Fc hybrid structure glycosylation. Linear Regression Analysis, ** P < 0.01. [file JGH-34-1878-s002.jpg]

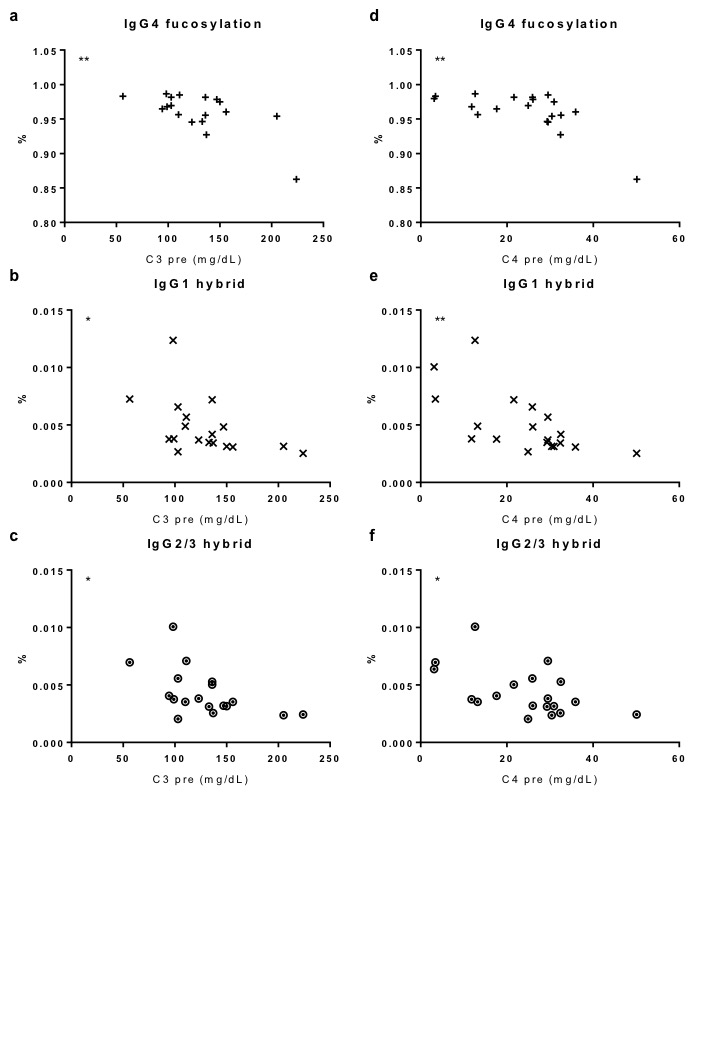

Supplement: Supplementary file 3 — Figure S3. Complement levels with glycosylation status in IgG4‐RD. Correlation plot of C3 levels with (a) fucosylation, and (b) IgG1 hybrid structures (c) IgG2/3 hybrid structures. Correlation plot of C4 levels with (d) fucosylation, and (e) IgG1 hybrid structures (f) IgG2/3 hybrid structures. Spearman rank correlation, * P < 0.05 ** P < 0.01. [file JGH-34-1878-s003.jpg]
